# Expression of CXCR3 on Adaptive and Innate Immune Cells Contributes Oviduct Pathology throughout *Chlamydia muridarum* Infection

**Published:** 2017-08-31

**Authors:** Janina Jiang, Heather Maxion, Cheryl I. Champion, Guangchao Liu, Kathleen A. Kelly

**Affiliations:** 1 Department of Pathology and Laboratory Medicine, David Geffen School of Medicine at UCLA, 10833 Le Conte Ave. CHS 1P-177, LA, CA 90095, USA; 2 California Nano Systems, University of California Los Angeles, Los Angeles, California, USA

**Keywords:** CXCR3, Immune cells, Adaptive immunity, Innate immunity, Oviduct pathology, *Chlamydia muridarum* infection

## Abstract

CXCR3 is a chemokine receptor expressed on a wide range of leukocytes, and it is involved in leukocyte migration throughout the blood and lymphatics. Specifically, CXCR3 is required for lymphocyte homing to the genital mucosa. When compared to wild type (WT) mice, CXCR3 deficiency (CXCR3−/−) mice infected with *Chlamydia muridarum (C. muridarum)* did not display impaired clearance and resolution of infection. However, they possessed significantly higher bacterial burden and lower levels of IFN-γ-producing TH1 cells. The knockouts also demonstrated a significant decrease in the level of activated conventional dendritic cells in the GT, ultimately leading to the decrease in activated TH1 cells. In addition, few activated plasmacytoid dendritic cells, which possess an inflammatory phenotype, were found in the lymph node of infected mice. This reduction in pDCs may be responsible for the decrease in neutrophils, which are acute inflammatory cells, in the CXCR3−/− mice. Due to the significantly reduced level of acute inflammation, these mice also possess a decrease in dilation and pathology in the oviduct. This demonstrates that the CXCR3−/− mice possess the ability to clear *C. muridarum* infections, but they do so without the increased inflammation and pathology in the GT.

## Introduction

*Chlamydia trachomatis*, an obligate intracellular bacterium, is the most prevalent sexually transmitted bacterial infection worldwide. Although antibiotic treatment is available, inflammation of the reproductive tract following chlamydial infection can cause severe complication, such as pelvic inflammatory disease and infertility. Chlamydial pathology is attributed to severe tissue inflammation leading to scarring and dysfunction. Currently, an effective vaccine against chlamydial infection is not available. Extensive efforts have been targeted to understanding chlamydial infection pathogenesis, as a guide for effective vaccine development and more efficient clinical therapy to prevent severe complications.

Investigations accessing the immune responses caused by chlamydial infection has indicated that T cells are central to clearance of infection [1]. Among T cell responses, IFN-γ secreting CD4 T cells (Th1 cells) are primary and required for clearance of chlamydial infection [2,3]. Although CD8 T cells and B cells may contribute to clearance of bacteria in primary infection, they are not required [4–6]. In addition, innate immune cells and cytokines [7–9] are also involved in immune responses and pathogenesis during chlamydial infection.

Chemokines are 8- to 10-kDa secreted proteins. Together with their receptors, they regulate migration and activation of not leukocytes, including the DC, but also of stromal cells [10]. CXCR3 is an inflammatory chemokine receptor whose expression is associated with Th1 CD4 T cells and CD8 cytotoxic lymphocytes [11]. CXCR3 is absent on naïve T cells, but is rapidly up-regulated following dendritic cell (DC)-induced T cell activation [12,13]. In addition, CXCR3 is also highly expressed on NK cells, plasmacytoid DCs, B cells and neutrophils [14–16] and direct those cells in the localization of first-line defenders at sites of infection and inflammation. CD4 T cell responses, especially Th1 cells, have been a primary defense force to eradicate bacteria and eliminate the infection. Although CXCR3 and its ligands are crucial for Th1 cells activation and migration, much of CXCR3’s role in chlamydial infection pathogenesis remains unexplored. In this study, we explored the impact of CXCR3 on modulation of host immune responses and infection course following *C. muridarum* intravaginal challenge using CXCR3−/− mice.

## Materials and Methods

### CXCR3 −/− mice

CXCR3 deficient (CXCR3−/−) mice on a Balb/C background (8 backcrosses) were a gift from Dr. Bao Lu (Department of Pediatrics, Children’s Hospital, Boston, MA). Age-matched WT (+/+) Balb/C mice controls were purchased from Harlan Sprague-Dawley (Indianapolis, IN). Animals were housed according to the American Association of Laboratory Animal Care guidelines and experimental procedures were approved by the UCLA Institutional Animal Care and Use Committee.

CXCR3 deficiency in CXCR3 −/− mice was confirmed by polymerase chain reaction. DNA from tail tips of 9 CXCR3 −/− mice and 1 WT mouse were amplified using the following forward and reverse primers: GCCTTCCTGCTGGCTTGTAT and TAGCTGCAGTACACGCAGAG. Genotyping was completed using the following optimized thermal cycling conditions: Denaturing at 94°C for 30s, annealing at 60°C for 30s, and extension at 72°C for 30s, all repeated for 35 cycles, and the final extension at 72°C for 10 min. PCR product was then visualized on a 3% Agarose gel (data not shown).

### Chlamydia preparation, infection and isolation of *Chlamydia muridarum* from cervical vaginal swabs

*Chlamydia muridarum* (Nigg) was grown, purified and titrated in McCoy cells as described previously [17]. Elementary bodies (EB) and reticulate bodies (RB) were isolated from McCoy cells and frozen at −80°C in sucrose-phosphate buffered saline (SPS) until use.

Mice 6 to 8 weeks of age were first injected subcutaneously with 2.5 mg of medroxyprogesterone acetate (DEPO PROVERA, Upjohn, Kalamazoo, MI) in 100 μl of sterile phosphate- buffered saline. Medroxyprogesterone drives mice into a state of anestrus, thus eliminating the variability in the rate and severity of infection due to the estrus cycle. Mice were inoculated with 1.5 × 10^5^ IFU of *C. muridarum* (Nigg strain) while under sodium pentobarbital 7 days later. Mice were sacrificed on day 7 after inoculation to assess innate and adaptive cell infiltrates to GT, Spl, and ILN, respectively, and on day 49 to assess oviduct pathology. Bacterial load was monitored by collecting cervical-vaginal swabs (Dacroswab Type I, Spectrum Labs, Houston, TX) every 3 days post-infection. Swabs were stored in sucrose phosphate buffer at −70°C until analyzed.

Swabs were prepared as previously described [18]. Individual wells of McCoy cell monolayers in 96 well-plates that were inoculated with 200 μl of the solution from the vaginal swabs, followed by centrifugation at 1,900 x g for 1 hour at 35°C. The plates were incubated for 2 h at 37°C. At this time the isolation solutions were removed, fresh cyclohexamine medium was added, and the plates were incubated for an additional 32 h. The cultures were fixed with methanol, and the Chlamydia inclusion bodies were identified by the addition of anti-MoPn immune sera and anti-mouse IgG conjugated to fluorescein isothiocyanate (ICN, Immunobiologicals, Irvine, CA). Inclusion bodies within 20 fields (x40) were counted under a fluorescence microscope, and numbers of IFU per milliliter were calculated. Mice were considered free from infection when no inclusion bodies were detected at two consecutive time points.

### Histological analysis

Oviduct pathology was analyzed 49 days post infection. Tissues were harvested, paraffin-embedded, and sectioned as described previously [19]. Sections were prepared and stained with either hematoxylin and eosin (H&E) or gomori trichrome. Slides were scanned at 20x and 40x magnification by the UCLA Translational Pathology Core Laboratory (TPCL) for analysis using ImageScope v 10.2 (Aperio Technologies). Sections stained with H&E were assessed for oviduct dilation, acute inflammation, and chronic inflammation. The diameter of each oviduct lumen was analyzed and measured by ImageScope, 6 mice per group were measured from H&E stained section collected transversally at the ovary to oviduct transition. Oviduct acute and chronic inflammation was evaluated semi-quantitatively with a score of 0 to 4, where 0 represents no inflammation and 4 represents high inflammation. Sections stained with gomori trichrome were assessed for oviduct fibrosis. The level of fibrosis was determined Sections stained with trichrome were evaluated using semi-quantitative scoring from 1 to 4 (1+ ≤ 25% light blue oviducts; 2+ ≥ 25% light blue oviducts; 3+ ≤ 25% dark blue oviducts; and 4+ ≥ 25% dark blue oviducts) [20–22].

### Isolation of lymphocytes for FACS staining and cell sorting

Lymphocytes were isolated from mice on the indicated day after infection. For Iliac lymph nodes (ILN) or the spleen (Spl), isolated tissue was meshed through 100 μm cell drainer (BD Falcon), individual cells were washed with 1% BSA in PBS followed by red blood cell lysis treatment. Isolation of lymphocytes from the genital tract (GT) was carried out as described [23–25]. Briefly, the entire GT was removed and minced into 0.5 mm pieces, which were then rinsed with Ca^2+^ Mg^2+^ -free Hanks’ balanced salt solution (HBSS). The tissue was incubated in a solution of 5 mM EDTA in HBSS at 37°C for two of 15 min with gentle stirring. Afterward, the tissue was incubated with RPMI 1640 containing 10% bovine calf serum, antibiotics, 25 mM HEPES and 1.5 mg/ml collagenase (Sigma, USA) at 37°C with stirring for two periods of 1 h. The isolated cells were pooled together, separated on a 40/75% discontinuous Percoll gradient (Pharmacia, Piscataway, N.J.) centrifuged at 2000 rpm at 22°C for 20 min. Mononuclear cell pellets were re-suspended in RPMI 1640 at 4°C until further use.

### Intracellular cytokine staining by Flow cytometry

Identification of cell subsets was done by fluorescence-activated cell sorting (FACS) as described previously [7,24]. Lymphocytes from GT, ILN and Spl were stimulated overnight with UV-inactivated EB. For intracellular cytokine staining, brefeldin A (Sigma) was added 6 h before the end of culture. For surface staining, cells were then stained with fluorochrome-labeled antibodies against CD3, CD4, CD8, DX5, CXCR3, CD19, CD11c, CD11b, CD45R, Gr-1, F4/80, CD69, CD80, CD9, and Siglec-H for 30 min at 4°C in dark. Cells then were treated with Fixation/Permeabilization (eBioscience, San Diego) for 1 h at 4°C in dark, followed by incubation with Fluorochrom-labeled IFN-γ/TNF-α antibodies for another 30 min under the same condition. Flow cytometry gating strategy: cells were first gated on FSC-A *vs.* FSC-H to identify single cells. Then lymphocytes, monocytes and neutrophils were gated by FSC and SSC. Further, cell types were identified by a combination of cell surface markers: CD4+ T cells (CD3+ CD4+), CD8+ T cells (CD3+ CD8+), Neutrophils (GR-1+ CD11b+), Macrophages (F4/80+ CD11b+), pDC (CD3− CD19− CD11c+ Gr-1+ CD45R+), cDC (CD3− CD19− CD11c+ CD11b+), NK (CD3− DX5+), NKT (CD3+ DX5+), CD69, CD80, CD9, Siglec-H were used as activation markers, IFN-γ, TNF-α were applied as function markers to identify certain populations. Fluorochrome or biotin conjugated antibodies were purchased from Biolegend (San Diego). FACS was performed on a LSR II digital analyzer (Becton Dickinson) at the UCLA Jonson Comprehensive Cancer Center and Center for AIDS Research Flow Cytometry Core Facility. FACS results were analyzed using Flowjo (Tree Star, Oregon).

### Statistical analysis

All statistical analysis was completed using GraphPad Prism version 5.04. Differences between WT (+/+) and CXCR3 −/− mice in *C. muridarum* burden were determined using two-way analysis of variance (ANOVA) with a Bonferoni post-hoc test comparing replicate means, and differences in course of infection were determined by the Mantel-Cox log-rank test. Comparison of the cell numbers between WT (+/+) and CXCR3 −/− mice on days 0 and 7 post infection in the Spl, ILN and GT were determined by one-way ANOVA with a Bonferoni post-hoc test. Differences in oviduct dilation and oviduct acute inflammation were compared using a Mann-Whitney test. Groups were scored statistically different at P<0.05.

## Results

### Lack of CXCR3 alters resolution of *C. muridarum* infection

**Figure 1:**
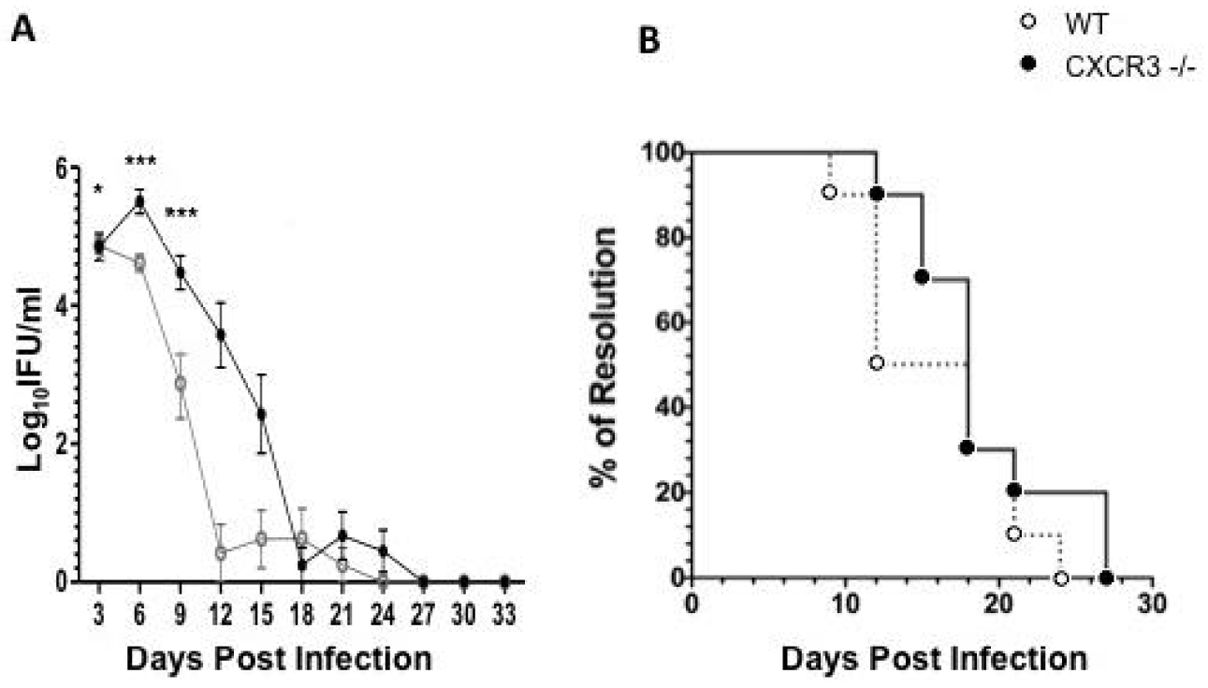
Bacterial burden during *C. muridarum* genital infection; A) *C. muridarum* was measured in vaginal swabs collected every three days post-inoculation. Groups were compared by two-way repeated measures-ANOVA, followed by Bonferonni post-hoc test. n=10; B) Percent of infected mice per group during the infection course was compared by log-rank (mantel-cox), n=10. Data is compiled from two independent experiments where each experiment represents 5 mice. **=p<0.01, ***=p<0.001.

In order to determine the ability of CXCR3−/− mice to clear infections of Chlamydia, wild-type and knockout mice were infected with *C. muridarum* (CM), and the bacterial burden of these mice were measured every three days post infection using vaginal swabs. Both the WT and CXCR3−/− mice cleared the CM infection by 27 days post infection; however, the knockout mice possessed a significantly higher bacterial burden on days 3, 6 and 9, when compared to WT mice (Figure 1A). Although the CXCR3−/− mice possessed a higher bacterial burden during the course of CM infection, no significant difference existed between the time courses of resolution of infection when compared to the WT mice (Figure 1B). This demonstrates that the CXCR3−/− mice can effectively resolve chlamydial infections, even though they possess a higher bacterial burden during infection course.

### CXCR3 KO mice display lessen UGT pathology

**Figure 2:**
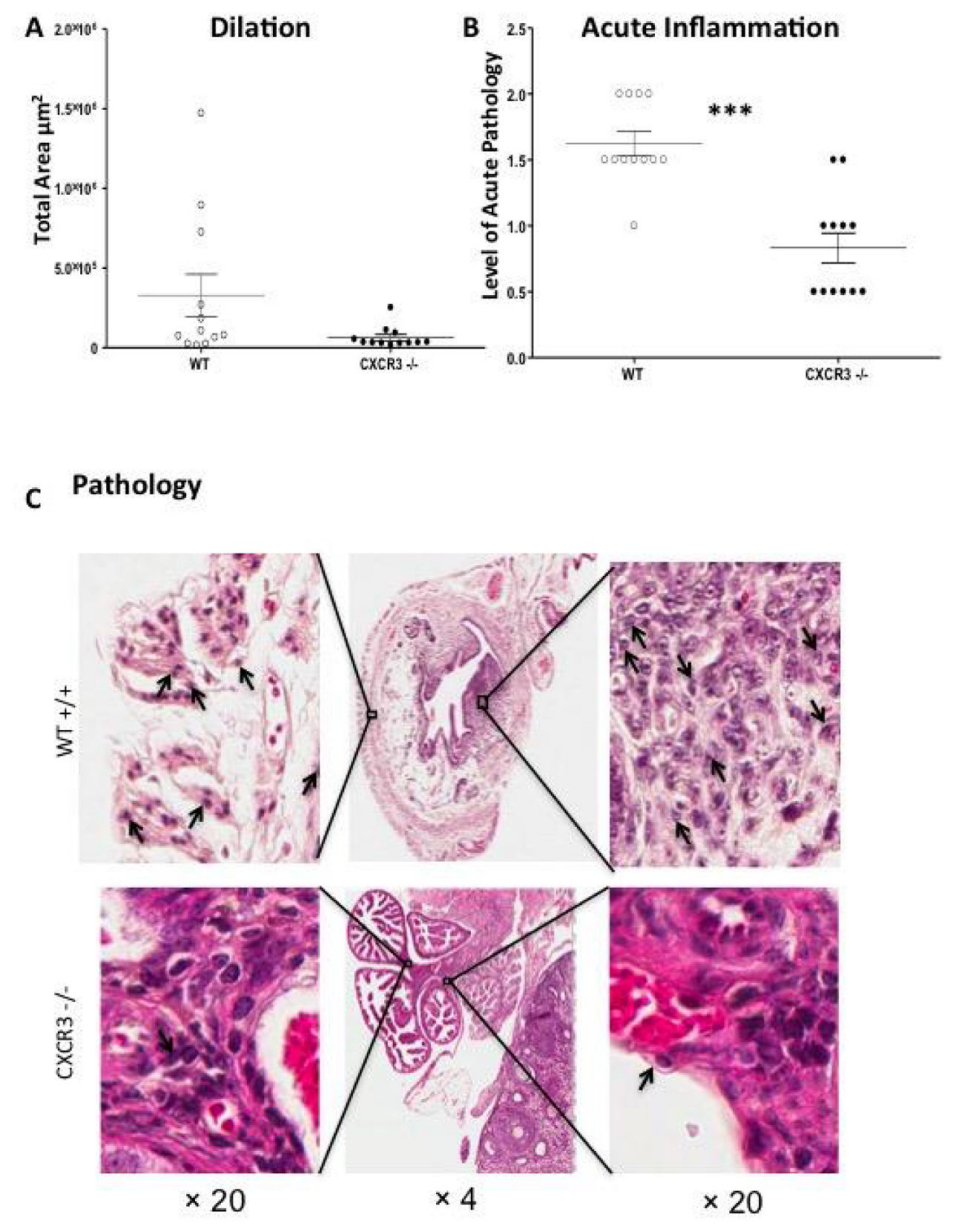
Oviduct histopathology during *C. muridarum* infection; A) Dilation of oviducts 49 days after infection. OD diameters of 6 mice per group were measured from H&E stained sections collected transversally at the ovary to OD transition; B) Data is expressed as acute inflammation. Inflammation was scored on a scale of 0 to 4 for individual mice based on a qualitative assessment. Individual points represent the scores for the left and right oviducts for a single mouse. Averages are shown ± SEM for 12 individual mice. (***, p<0.001, * p<0.05) by Mann-Whitney test; C) Oviduct pathology in a WT and CXCR3−/− mouse. Neutrophils are indicated by arrows.

We evaluated UGT (uterine horns and oviducts) pathology in CXCR3−/− mice as well as WT counterparts by examining inflammatory scores in the UGT from hematoxylin and eosin stained slides and measuring the dilation of oviducts. GT tissues were harvested 49 days after genital infection with *C. muridarum*. Our data showed that CXCR3−/− mice had decreased oviduct dilation (Figure 2A), in comparison with and significantly lower oviduct diameter measurement than WT. Furthermore, acute inflammation scores on a scale of 0 to 4 for individual mice based on a qualitative assessment revealed (Figure 2B) that CXCR3−/− mice with an average score of 0.8 were remarkably lower than WT 1.6. In addition, oviduct pathology (Figure 2C) indicated that CXCR3−/− mice had less neutrophil infiltration than WT mice. Together, our data suggest that chemokine CXCR3 is involved in tissue pathology and damage in mouse genital tract after *C. muridarum* intravaginal infection.

### Impaired IFN-γ+ TH1 responses and altered functional CD8 subsets are shown in CXCR3−/− mice during *C. muridarum* infection

**Figure 3:**
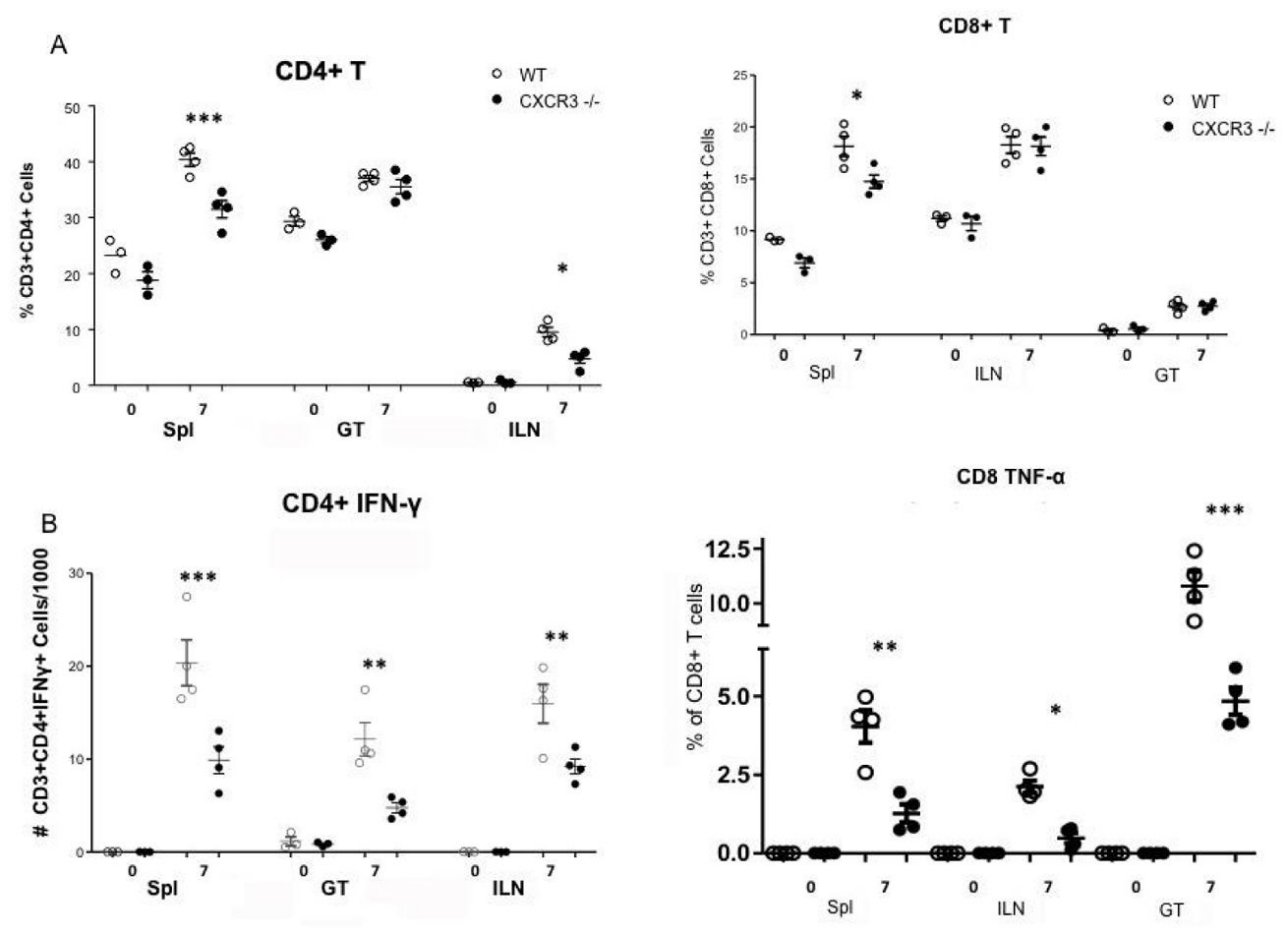
T cells influx during *C. muridarum* genital infection. Mice were infected as described for Figure 1. The spl. iLN and genital tract were harvested from WT and CXCR3−/− mice 0 and 7 days post infection. Single cell suspensions were labeled with anti-CD3, CD4, CD8, IFN-γ and TNF-α. In all cases, a minimum of 106 events were acquired on the flow cytometer. Each data point represents a pool of four mice comparing WT and CXCR3 −/− using Prism one-way ANOVA with a Bonferonni Post-test, n=4. (*, p<0.05)(**, p<0.01, ***, p<0.001); A) CD4+ T cells; B) The number of activated Th1 cells; C) CD8+ T cells; D) CD8+TNF-α cells.

Since the bacterial burden of the CXCR3−/− mice was significantly higher during the infection with Chlamydia comparing with WT, we looked further into adaptive immune responses elicited in these two strains. On day 7 post intravaginal infection with CM, lymphocytes were collected from two strains of mice Spls, iLNs and GTs, as mentioned in Methods. Cells were preceded for surface staining as well as intracellular cytokine staining for IFN-, TNF-α. As shown in (Figure 3A), our data indicated that on day 7 post infection, CXCR3−/− mice had significantly less amount of CD4 T cells in Spl and iLN, but not in GT, than WT. The number of IFN-γ+ CD4 T cells: TH1 cells, the lymphocytes primarily responsible for clearing chlamydial infections [7,26,27], was measured by FACS in the spleen (Spl), iliac lymph node (ILN), and the genital tract (GT) 7 days post infection. The CXCR3−/− mice possessed significantly lower levels of activated TH1 cells in the spl, ILN, and the GT on day 7 (Figure 3B). On the other hand, different levels of CD8 T cell between two strains were only observed in spl, not in GT or iLN, with a decrease seen in CXCR3−/− (Figure 3C). Furthermore, functional subset of CD8 T cells (TNF-a+ secreting CD8 T cells) was significantly lower in CXCR3−/− at three locations checked than WT as shown in Figure 3. The most profound increase was observed in the genital tract, where tissue histopathology is formed post infection. CD8+TNF-a+ has been strongly link to tissue damage and pathology [7,28]. Our findings indicate that CXCR3 involves in CD8+TNF-a+ activation and contributes to tissue damage.

### Deficiency of CXCR3 blocks dendritic cells (DCs) influx to the infection sites

**Figure 4:**
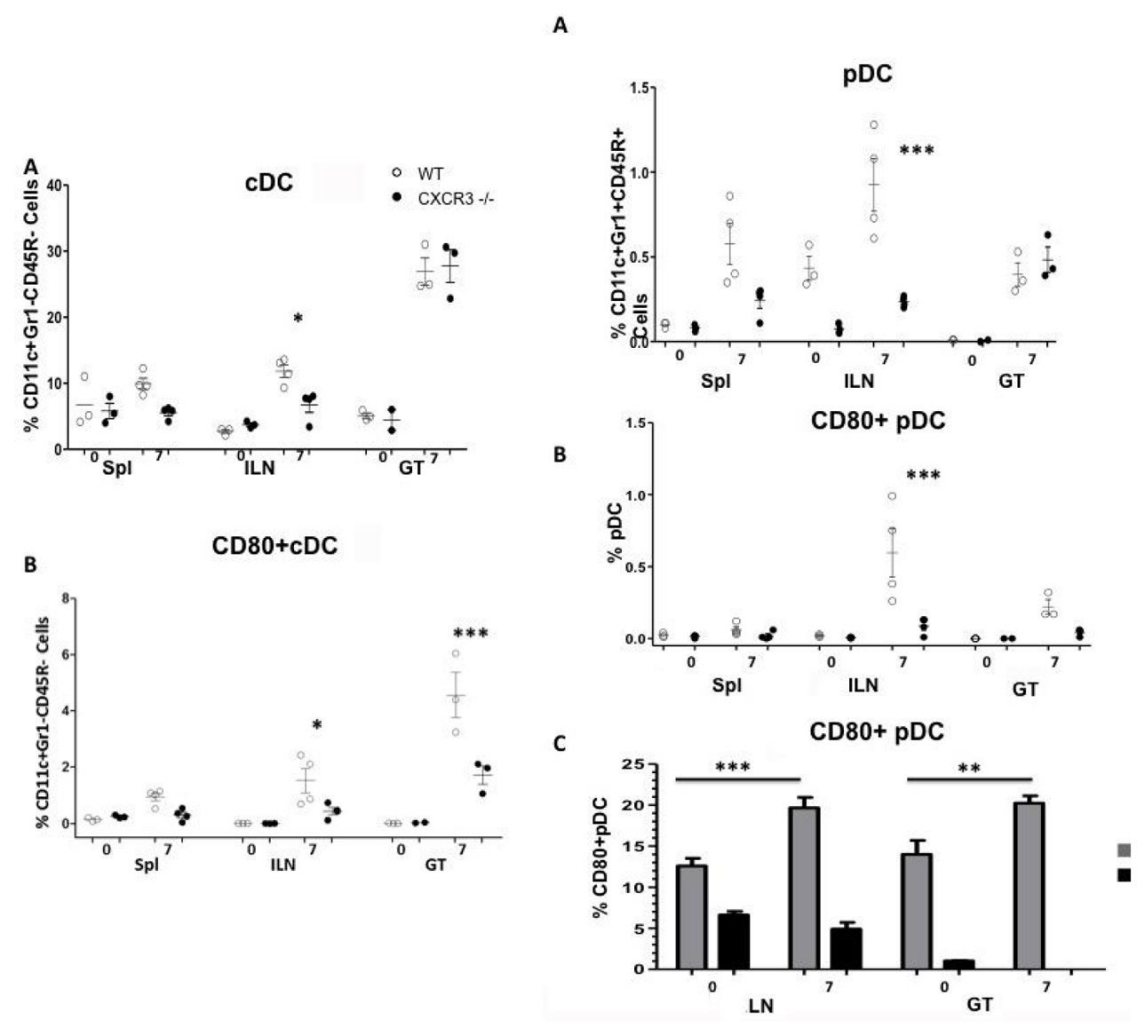
Dendritic cells influx during *C. muridarum* genital infection. Lymphocytes were isolated from various locations on D0 and D7 post challenge. Cells were analyzed for surface markers by FACS; A) cDC; B) Activated cDC; C) pDC; D) Activated pDC; E) CD9 and Siglec-H CD9 and Siglec-H expression in the iLN and GT in WT mice 0 and 7 days post infection. Each data point represents a pool of three mice comparing WT and CXCR3 −/− using one-way ANOVA with a Bonferonni Post-test, n=4. (*, p<0.05)(***, p<0.001).

Antigen presenting cells, such as dendritic cells, play a pivotal role in processing antigens, presenting to T cells and eliciting, and directing adaptive immune responses. In order to access how CXCR3 deficiency affects dendritic cells function in the event of chlamydial infection, we infected CXCR3−/− and WT mice intravaginally. 7 days later, we dissected DC subsets (classic DC, cDC and plasmacytoid DC, [pDC]) quality and quantity by FACS. Our data revealed that with lack of CXCR3, mice had less cDC in iLN post infection, in comparison with WT, but in Spl and GT, there is no difference. Furthermore, activated cDC, with expression of CD80, was less in knockout mice than WT. This decrease was shown in iLN, and more profoundly in GT (Figure 4A and 4B). In compartment of pDC, Our data revealed that CXCR3−/− had less amount of pDC, as well as activated pDC (CD80+ pDC) in iLN, but no difference was observed in spl and GT between two strains of mice (Figure 4C and 4D). pDC can be further phenotypically and functionally classified into two subsets: one expressing CD9+ secreting IFN-α (pro-inflammatory), another with Siglec-H+ pDC (suppressive) induces Foxp3+ CD4+ T cells and suppresses antitumor immunity [29,30]. To further shed light on the roles of pDC subsets on chlamydial infection, we infected wild type mice intravaginally with *C. muridarum*, on day 7 post infection, we accessed activated pDC profile from local draining lymph nodes: iLN and infection site: GT by FACS. As shown in (Figure 4E), our data indicated that under normal condition (D0), pDC in iLN and GT consists of both pro-inflammatory and suppressive subsets, with a dominance of CD9+ CD80− +pDC. After infection (D7), CD9+ subset was significantly increased (compared with D0) in both iLN and GT, of note suppressive pDC (Siglec-H+) was under detective level in GT post infection, suggest that host response to invading pathogens with increased pro-inflammatory pDC, further initiate protective immunity; also with decrease suppressive pDC, which unlikely to benefit host fight against infection.

### CXCR3 also alters leukocytes influx during *C. muridarum* infection

**Figure 5:**
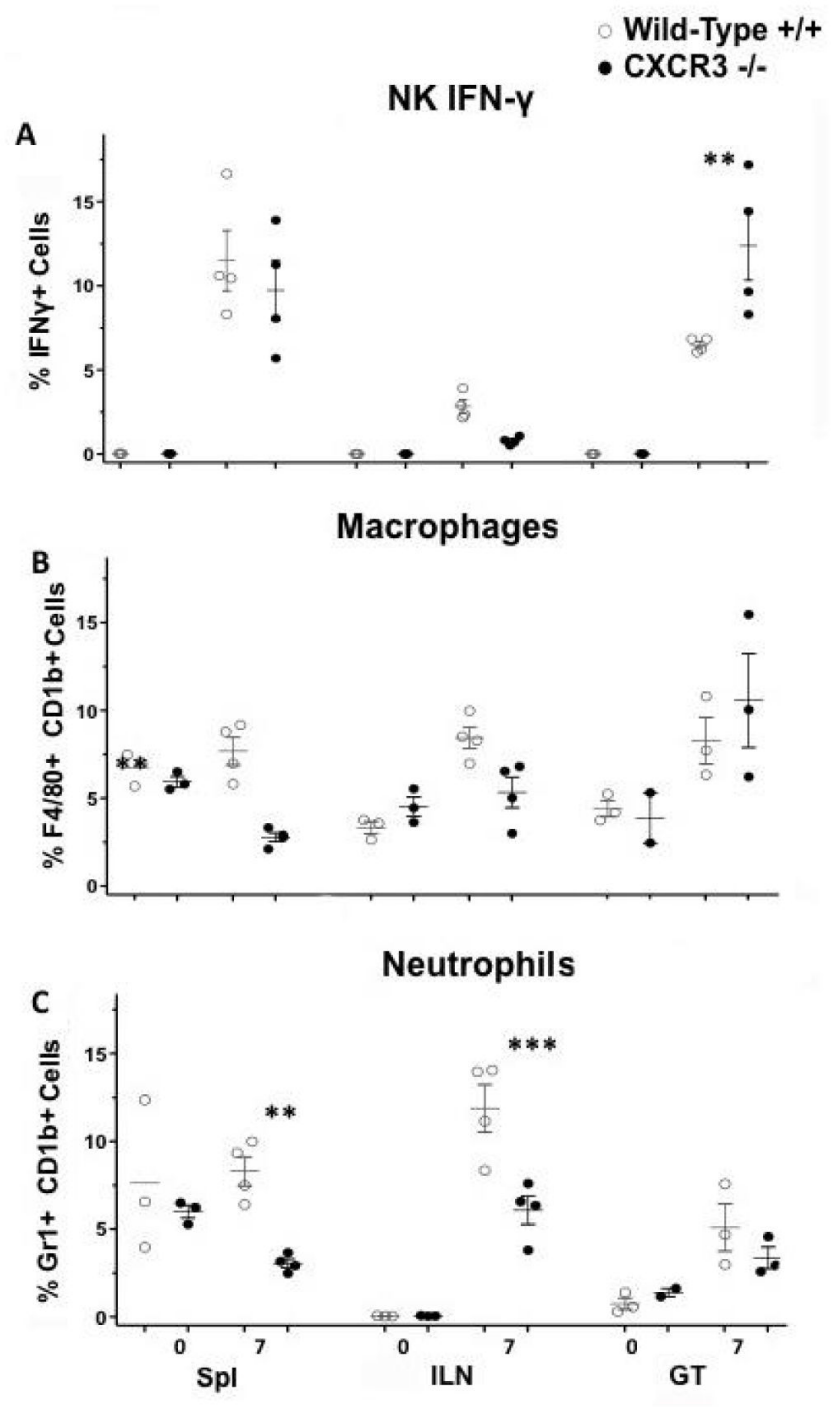
Leukocyte influx during *C. muridarum* genital infection. Mice were challenged intravaginally with *C. muridarum*; A) IFN-γ-producing CD8+ T Cell; B) IFN-γ-producing NK Cell; C) Macrophage; Neutrophil influx to Spl, ILN, and GT in WT and CXCR3−/− mice 0 and 7 days post infection. Each data point represents a pool of three mice comparing WT and CXCR3 −/− using Prism one-way ANOVA with a Bonferonni Post-test, n=4. (*, p<0.05)(**, p<0.01) (***, p<0.001).

In order to examine CXCR3’s influences on leukocyte functions during CM infection, we infected both CXCR3−/− and WT counterpart intravaginally, and identified IFN-γ secreting NK cells, macrophages, and neutrophils by flow cytometry. Our data (Figures 5A–5C) disclosed that CXCR3−/− had higher level of NK IFN-γ and macrophage in the genital tract in comparison with WT. This was not observed in other locations (Spl, iLN). However, CXCR3−/− had less neutrophils in spl and iLN but not in GT, in contrast to WT. These findings suggest that CXCR3 plays a role in the NK cell, macrophage and neutrophil activation, homing and functions during *C. muriduram* genital infection.

Taking together, our research demonstrates that chemokine receptor CXCR3 involves a variety of immune cells activation and trafficking in chlamydial infection mouse model. Following chlamydial genital infection, CXCR3 activates cDC and pDC, and directs them to the infection site and activates CD4 and CD8 T cells into functional subsets, including (not only) Th1 cells, CD8TFN-α T cells. In addition, CXCR3 also activates and regulates NK cells, macrophages, neutrophils. As a result, CXCR3−/− mice resolved bacterial infection less efficient than WT, but they had lessened tissue damage.

## Discussion

In order to clear invading pathogens, immune cells have to be able to traffic to infection sites. Chemokine receptors are pivotal for directing lymphocytes to their destinations. CXCR3, through binding of its chemokine ligands, CXCL9 (also known as monokine induced by IFN-γ, MIG); CXCL10 (interferon-induced protein of 10 kDa, IP-10); CXCL11 (interferon-inducible T cell alpha chemoattractant, I-TAC) [11,31] has been shown to induce migration and coordinate inflammation in the periphery. The CXCR3 and its ligands represent a complex chemokine system: one receptor has three ligands, like other chemokine-chemokine receptor, they have been shown work redundantly, collaboratively. The mechanism or reason of this redundancy remains unknown and deserves further investigation.

In this study, we demonstrated that the lack of CXCR3 interferes with the ability of mice to control genital infection with *C. muridarum*. Even though CXCR3−/− mice eventually are able to clear up infection in time as WT, there is no overall delay for CXCR3−/− mice to clear a vaginal infection compared to WT. Most likely, the lack of chemokine receptors affects the activation and homing of Th1 CD4 T cells to genital track, which are pivotal for eliminating infection. This has been confirmed by others [32]. Through all the locations checked, Th1 T cells, the cell type responsible for clearance of infection, were significantly lower in CXCR3−/− mice than in WT.

Our data also indicate that CXCR3 expressed on other cell types associated to IFN-γ production also influences infection. Lack of CXCR3 affects activation and homing of cDC, results in less CD80 cDCs present in GT. Since cDC are the major resource for polarizing naïve T cell towards to Th1 cells [8,33], the reduced cDC numbers in iLN results in further Th1 cell deficit seen in CXCR3−/− mice. Together, this leads us conclude that CXCR3 is more profoundly involved in Th1 cell activation and homing than total CD4 T cell. The latter was only shown difference in iLN between two strains, but Th1 cell was shown is multiple locations. Our observation is in agreement with others [11,34,35]. Although CXCR3−/− overtime eradicate infection is at the same pace as WT, this might be due to the contribution of another major IFN- resource: NK cells [36,37]. We show that more IFN- secreting NK cells are detected in GT of CXCR3−/− than of WT, which likely contributes to the compensation of CXCR3 knockout mice in cleaning infection.

The role of pDC in chlamydial infection has been controversial. Some studies have suggested that pDC is more potent to induce pro-inflammatory cytokines and modulate non-protective T-cell responses [19,38]; Others indicate that in Chlamydia pneumonia lung infection model, depletion of pDCs increased the severity of infection and lung pathology [39]. These controversies might come from different bacterial strains used, and different disease model and locations investigated. In this study, we focused on the impact CXCR3 had on pDC functions and subsequent influence on immune responses. Here we demonstrate pDC interacts with T cells in local draining nodes, it activate and differentiate T cells, guide them to infection sites. Less pDCs in CXCR3−/− may partially contribute to protective adaptive immune responses and high bacterial load observed in knockout mice. In addition, less pDC in CXCR3−/− mice may also contribute to less pathology and tissue damage observed in knockout mice oviducts, which is in agreement with other’s reports [8,33].

Plasmacytoid dendritic cells have been further classified into two subsets with distinctly different functions [29], one with CD90 expression, which is the main source of IFN-α. IFN-α, in turn, can activate NK cells, promote CTL activity, induces B cell, Ab production and different myeloid DC [39–44]. This subset is also called pro-inflammatory pDC. Another subset, with Siglec-H expression, which promotes Ag-specific regulatory T cell activation and induces tolerance, is also called suppressive pDC. Interestingly, chlamydial infection increases CD90pDC and decreases Siglec-H pDCs, and suggests hosts promote pro-inflammatory pDC when confronting with a pathogen challenge in order to control infection. It would be interesting to see how a lack of CXCR3 would affect pDC subset profile, which is currently under investigation.

*Chlamydia trachomatis* genital infection can lead to immune-mediated damage of the female reproductive organs and serious reproductive disability: PID and infertility. Although female infection is easily detected and treated with antibiotics, treated individuals can acquire repeated infection. Repeated inflammation is implicated as a cause of PID and infertility [45]. Although the cause of PID and infertility remain unknown, studies have shown certain immune cells are involved. CXCR3 is associated with CD8 cytotoxic T cells [11,34]. In our study, we also show that CXCR3 regulates CD8 and CD8+TNF-a+ cell migration after infection. Interestingly, we and others have shown CD8+TNF-a+ cells are responsible for tissue damage and pathology [45]. Deficiency of CXCR3 leads to less CD8+TNF-a+ present in GT, spl and iLN, which is related to lessened pathology seen in knockout mouse GT tissue. In addition, neutrophils, which also have been shown associated to tissue damage, are impaired because of inadequate CXCR3 expression. Together, our study suggests CXCR3 regulate CD8, CD8+TNF-a+ and neutrophils, those cells are involved or partially involved in inducing tissue pathology following chlamydial infection.

In summary, this first report reveals that CXCR3 not only regulates Th1 T cell activation and trafficking, but also modulates CD8+TNF-a+, cDC, pDC, neutrophil and NK cells in chlamydial infection. Our results highlight the diverse roles of CXCR3 in chlamydial infection: it regulates and enhances host defense mechanisms against infection, and also contributes to tissue inflammation and pathology. It would be a crucial key to balance the advantages and disadvantages elicited by CXCR3 and other chemokine receptors when to develop efficient treatments and a successful chlamydial vaccine.

## References

[1] Brunham RC, Rey-Ladino J (2005). Immunology of Chlamydia infection: implications for a Chlamydia trachomatis vaccine. Nat Rev Immunol.

[2] Darville T, Hiltke TJ (2010). Pathogenesis of Genital Tract Disease Due to Chlamydia trachomatis. J Infect Dis.

[3] Igietseme Joseph U, He Qing, Joseph Kahaliah, Eko Francis O, Lyn Deborah (2009). Role of T Lymphocytes in the Pathogenesis of Chlamydia Disease. J Inf Dis.

[4] Nogueira CV, Zhang X, Giovannone N, Sennott EL, Starnbach MN (2015). Protective immunity against Chlamydia trachomatis can engage both CD4+ and CD8+ T cells and bridge the respiratory and genital mucosae. J Immunol.

[5] Magee DM, Williams DM, Smith JG, Bleicker CA, Grubbs BG (1995). Role of CD8 T cells in primary Chlamydia infection. Infect Immun.

[6] Su H, Feilzer K, Caldwell HD, Morrison RP (1997). Chlamydia trachomatis genital tract infection of antibody-deficient gene knockout mice. Infect Immun.

[7] Jiang J, Karimi O, Ouburg S, Championet CI, Khurana A (2012). Interruption of the CXCL13-CXCR5 axis increases upper genital gract pathology following chlamydial genital infection. PLos ONE.

[8] Moniz RJ, Chan AM, Kelly KA (2009). Identification of dendritic cell subsets responding to genital infection by Chlamydia muridarum. FEMS Immunol Med Microbiol.

[9] Rasmussen SJ, Eckmann L, Quayle AJ, Shen L, Zhang YX (1997). Secretion of proinflammatory cytokines by epithelial cells in response to Chlamydia infection suggests a central role for epithelial cells in chlamydial pathogenesis. J Clin Invest.

[10] Baggiolini M, Dewald B, Moser B (1994). Interleukin-8 and related chemotactic cytokines -cxc and cc chemokines. Adv Immunol.

[11] Loetscher M, Gerber B, Loetscher P, Jones SA, Piali L (1996). Chemokine receptor specific for IP10 and Mig: Structure, function, and expression in activated T-lymphocytes. J Exp Med.

[12] Xie JH, Nomura N, Lu M, Chen SL, Koch GE (2003). Antibody-mediated blockade of the CXCR3 chemokine receptor results in diminished recruitment of T helper 1 cells into sites of inflammation. J Leukoc Biol.

[13] Khan IA, MacLean JA, Lee FS, Casciotti L, DeHaan E (2000). IP-10 is critical for effector T cell trafficking and host survival in Toxoplasma gondii infection. Immunity.

[14] Thomas SY, Hou R, Boyson JE, Means TK, Hess C (2003). CD1d-restricted NKT cells express a chemokine receptor profile indicative of Th1-type inflammatory homing cells. J Immunol.

[15] Cella M, Jarrossay D, Facchetti F, Alebardi O, Nakajima H (1999). Plasmacytoid monocytes migrate to inflamed lymph nodes and produce large amounts of type I interferon. Nat Med.

[16] Nanki T, Takada K, Komano Y, Morio T, Kanegane H (2009). Chemokine receptor expression and functional effects of chemokines on B cells: implication in the pathogenesis of rheumatoid arthritis. Arthritis Res Ther.

[17] Maxion HK, Liu W, Chang MH, Kelly KA (2004). The infecting dose of Chlamydia muridarum modulates the innate immune response and ascending infection. Infect Immun.

[18] Kelly KA, Robinson EA, Rank RG (1996). Initial route of antigen administration alters the T-cell cytokine profile produced in response to the mouse pneumonitis biovar of Chlamydia trachomatis following genital infection. Infect Immun.

[19] Moniz RJ, Chan AM, Gordon LK, Braun J, Arditi M (2010). Plasmacytoid dendritic cells modulate nonprotective T-cell responses to genital infection by Chlamydia muridarum. FEMS Immunol Med Microbiol.

[20] Dahab GM, Kheriza MM, El-Beltagi HM, Fouda AM, El-Din OA (2004). Digital quantification of fibrosis in liver biopsy sections: descriptions of a new method by Photoshop software. J Gastroenterol Hepatol.

[21] Krajewska M, Smith LH, Rong J, Huang X, Hyer Marc L (2009). Image Analysis Algorithms for Immunohistochemical Assessment of Cell Death Events and Fibrosis in Tissue Sections. J Histochem Cytochem.

[22] Shimazaki K, Chan AM, Moniz RJ, Wadehra M, Nagy A (2009). Blockade of epithelial membrane protein 2 (EMP2) abrogates infection of Chlamydia muridarum murine genital infection model. FEMS Immunol Med Microbiol.

[23] Jiang J, Kelly KA (2012). Isolation of lymphocytes from mouse genital tract mucosa. J Vis Exp.

[24] Jiang JQ, He XS, Feng N, Greenberg HB (2008). Qualitative and quantitative characteristics of rotavirus-specific CD8 T cells vary depending on the route of infection. J Virol.

[25] Jiang JQ, Patrick A, Moss RB, Rosenthal KL (2005). CD8+ T-cell-mediated cross-clade protection in the genital tract following intranasal immunization with inactivated human immunodeficiency virus antigen plus CpG oligodeoxynucleotides. J Virol.

[26] Yu H, Jiang X, Shen C, Karunakaran KP, Jiang J, Brunham RC (2010). Chlamydia muridarum T-Cell Antigens Formulated with the Adjuvant DDA/TDB Induce Immunity against Infection That Correlates with a High Frequency of Gamma Interferon (IFN-{gamma})/Tumor Necrosis Factor Alpha and IFN-{gamma}/Interleukin-17 Double-Positive CD4+ T Cells. Infect Immun.

[27] Barral R, Desai R, Zheng X, Frazer LC, Sucato GS (2014). Frequency of Chlamydia trachomatis-specific T cell interferon-γ and interleukin-17 responses in CD4-enriched peripheral blood mononuclear cells of sexually active adolescent females. J Reprod Immunol.

[28] Rhodes KA, Andrew EM, Newton DJ, Tramonti D, Carding SR (2008). A subset of IL-10-producing gammadelta T cells protect the liver from Listeria-elicited, CD8(+) T cell-mediated injury. Eur J Immunol.

[29] Björck P, Leong HX, Engleman EG (2011). Plasmacytoid Dendritic Cell Dichotomy: Identification of IFN-α Producing Cells as a Phenotypically and Functionally Distinct Subset. J Immunol.

[30] Terlizzi M, Popolo A, Pinto A, Sorrentino R (2014). Plasmacytoid dendritic cells contribute to doxorubicin-induced tumor arrest in a mouse model of pulmonary metastasis. J Immunother.

[31] Cole KE, Strick CA, Paradis TJ, Ogborne KT, Loetscher M (1998). Interferon-inducible T cell alpha chemoattractant (I-TAC): a novel non-ELR CXC chemokine with potent activity on activated T cells through selective high affinity binding to CXCR3. J Exp Med.

[32] Olive AJ, Gondek DC, Starnbach MN (2010). CXCR3 and CCR5 are both required for T cell-mediated protection against C. trachomatis infection in the murine genital mucosa. Mucosal Immunol.

[33] Agrawal T, Vats V, Salhan S, Mittal A (2009). Determination of chlamydial load and immune parameters in asymptomatic, symptomatic and infertile women. FEMS Immunol Med Microbiol.

[34] Kim CH, Rott L, Kunkel EJ, Genovese MC, Andrew DP (2001). Rules of chemokine receptor association with T cell polarization in vivo. J Clin Invest.

[35] Yamamoto J, Adachi Y, Onoue Y, Adachi YS, Okabe Y (2000). Differential expression of the chemokine receptors by the Th1- and Th2-type effector populations within circulating CD4+ T cells. J Leukoc Biol.

[36] Tseng CK, Rank RG (1998). Role of NK cells in the early host response to chlamydial genital infection. Infect Immun.

[37] Nagarajan UM, Sikes J, Prantner D, Andrews CW, Frazer L (2011). MyD88 deficiency leads to decreased NK cell gamma interferon production and T cell recruitment during Chlamydia muridarum genital tract infection, but a predominant Th1 response and enhanced monocytic inflammation are associated with infection resolution. Infect Immun.

[38] Crother TR, Ma J, Jupelli M, Chiba N, Chen S (2012). Plasmacytoid dendritic cells play a role for effective innate immune responses during Chlamydia pneumoniae infection in mice. PLoS One.

[39] Joyee AG, Yang X (2013). Plasmacytoid dendritic cells mediate the regulation of inflammatory type T cell response for optimal immunity against respiratory Chlamydia pneumoniae infection. PLoS One.

[40] Liu YJ (2005). IPC: professional type 1 interferon-producing cells and plasmacytoid dendritic cell precursors. Annu Rev Immunol.

[41] Bjorck P (2001). Isolation and characterization of plasmacytoid dendritic cells from Flt3 ligand and granulocyte-macrophage colony-stimulating factor-treated mice. Blood.

[42] Asselin-Paturel C, Boonstra A, Dalod M, Durand I, Yessaad N (2001). Mouse type I IFN-producing cells are immature APCs with plasmacytoid morphology. Nat Immunol.

[43] Colonna M, Trinchieri G, Liu YJ (2004). Plasmacytoid dendritic cells in immunity. Nat Immunol.

[44] Burstein GR, Gaydos CA, Diener-West M, Howell MR, Zenilman JM (1998). Incident Chlamydia trachomatis infections among inner-city adolescent females. JAMA.

[45] Murthy AK, Li W, Chaganty BK, Kamalakaran S, Guentzel MN (2011). Tumor necrosis factor alpha production from CD8+ T cells mediates oviduct pathological sequelae following primary genital Chlamydia muridarum infection. Infect Immun.

